# Development and Validation of a PCR-ELISA for the Diagnosis of Symptomatic and Asymptomatic Infection by* Leishmania (Leishmania) infantum*

**DOI:** 10.1155/2017/7364854

**Published:** 2017-01-09

**Authors:** Fernanda Alvarenga Cardoso Medeiros, Luciana Inácia Gomes, Edward Oliveira, Carolina Senra Alves de Souza, Maria Vitória Mourão, Gláucia Fernandes Cota, Letícia Helena dos Santos Marques, Mariângela Carneiro, Ana Rabello

**Affiliations:** ^1^Centro de Pesquisas René Rachou, Oswaldo Cruz Foundation (Fiocruz), Belo Horizonte, MG, Brazil; ^2^Departamento de Parasitologia, Instituto de Ciências Biológicas, Universidade Federal de Minas Gerais, Belo Horizonte, MG, Brazil

## Abstract

A* kDNA* PCR enzyme-linked immunosorbent assay (*kDNA* PCR-ELISA) for the diagnosis of human visceral leishmaniasis (HVL) was developed. The detection limit of the reaction, precision measurements, and cut-off of the* kDNA* PCR-ELISA were defined in a proof-of-concept phase. A reference strain of* Leishmania (Leishmania) infantum* and a bank of 14 peripheral blood samples from immunocompetent patients with VL were characterized using techniques considered gold standards, and 11 blood samples obtained from healthy individuals of an endemic area were also assessed. Phase II evaluation determined the performance of the assay in peripheral blood samples from 105 patients with VL (adults and children), 25 patients with* Leishmania*/HIV coinfection, 40 healthy individuals, and 33 asymptomatic individuals living in endemic areas. The* kDNA *PCR-ELISA exhibited satisfactory precision, with a detection limit of 0.07 fg of DNA from* L*.* (L*.*) infantum* and 1 parasite/mL blood. The overall sensitivity of the assay for all groups studied was 100% (95% confidence interval [CI]: 97.1–100%), and the specificity was 95% (95% CI: 83.5–98.6%). The* kDNA* PCR-ELISA was shown to be a useful tool for VL symptomatic and asymptomatic individuals diagnosis and its use in endemic countries may help monitor control interventions.

## 1. Introduction

Visceral leishmaniasis (VL) is a major public health problem and has an estimated annual incidence of approximately 0.2 to 0.4 million cases and a focal distribution, with more than 90% of global cases occurring in six countries: India, Bangladesh, Sudan, South Sudan, Brazil, and Ethiopia [1]. In Brazil, the disease is an endemic anthropozoonosis caused by the parasitic protozoa* Leishmania *(*Leishmania*)* infantum*, which is an obligate intracellular protozoan belonging to the* Leishmania donovani *complex.* L. infantum* is primarily transmitted by the sand fly* Lutzomyia longipalpis*, and dogs are the main reservoir of these parasites. The number of cases has increased in large Brazilian cities despite Brazilian measures to control VL [2, 3]. A mean of 3.771 cases are detected annually in Brazil [4].

A noninvasive easily implemented high performance test for the detection of symptomatic and asymptomatic infection is lacking despite several technological advances in recent years. Parasitological examination, which is a reference technique, requires invasive sample collection from the spleen or bone marrow, which must be performed by trained professionals and often exhibits low and variable sensitivity. Antibody-based tests, such as immunofluorescence antibody test (IFAT), enzyme-linked immunosorbent assay (ELISA), and recombinant antigen-based immunochromatography test (ICT), support the clinical diagnosis of VL, but these methods have drawbacks, such as cross-reactions in the presence of other diseases and an inability to distinguish between active and past infection. Some studies demonstrated that serological tests based on antigens of a single* Leishmania* sp. exhibited low diagnostic performance in regions with circulating heterologous parasites [5, 6]. The serological tests, with the exception of the Direct Agglutination Tests (DAT) and Western blotting, exhibit low sensitivity in VL patients coinfected with HIV [7–9] because of their insufficient immune humoral response.

Molecular methods are useful for the detection of low parasitic loads and exhibit high sensitivity in the diagnosis of VL [10]. PCR-based assays are the most commonly used approaches in molecular diagnostics. One additional advantage of PCR is that it is based on the identification of conserved DNA sequences, which allows the detection of specific pathogen species in a variety of clinical samples, even archived materials [11]. PCR sensitivity is directly related to the copy number of the target and the primer and cycling protocol used [12]. The internal transcribed spacers (ITS, ITS1, and ITS2) regions, gp63 gene, HSP-70 gene, miniexon (ME), and SSU-rRNA and conserved and variable regions of kinetoplast DNA (kDNA) minicircles are used for the diagnosis and characterization of* Leishmania* strains [13]. Conventional PCR techniques targeting kDNA exhibit high sensitivity for VL diagnosis [13, 14], but this technique is not automated and is laborious, which limits its use to a small number of samples. To overcome this limitation, we developed a* kDNA* PCR-ELISA, which allows simultaneous testing of a large number of blood samples without the use of commercial kits for the detection of PCR products via measurement of absorbance readings for the laboratory diagnosis of VL.

## 2. Materials and Methods

This work was performed in two phases:

- In a proof-of-concept phase, the assay was standardized using a reference strain of* L*. (*L*.)* infantum* and a bank of peripheral blood samples from nonimmunosuppressed patients with VL that were characterized using techniques considered gold standards and from healthy individuals in an endemic area.
- In a phase II evaluation, the assay was validated in clinical samples from varied groups of VL patients of different ages and in asymptomatic and noninfected individuals to certify the wide use of the assay for the diagnosis of human VL. We describe the step-by-step development and internal validation of the* kDNA *PCR-ELISA below.

### 2.1. Parasites

Promastigotes of* L. (L.) infantum* (MHOM/BR/2002/LPC-RPV),* L. (L.) donovani* (MHOM/ET/196/HU3),* L*.* (Viannia) braziliensis* (MHOM/BR/75/M2903),* L*.* (V.) guyanensis* (MHOM/BR/1975/M4147), and* L*.* (L.) amazonensis* (IFLA/BR/1967/PH-8) were cultured in Schneider medium (Sigma-Aldrich, St. Louis, MO, USA) supplemented with 10% foetal bovine serum (Invitrogen, Carlsbad, CA, USA) and counted in a Neubauer chamber. An aliquot of 1 mL of the suspension of parasites was centrifuged at 2.300*g* for 2 minutes and stored at −70°C.

### 2.2. Samples

#### 2.2.1. Peripheral Blood Samples

Peripheral blood samples collected from 14 patients with VL, nonimmunosuppressed, living in endemic areas in Brazil, with typical VL symptoms and parasitological confirmation (direct exam and/or culture) with a mean age of 12.7 years (range one month to 76.8 years) were used as positive controls.

Peripheral blood samples from 11 healthy individuals from the endemic area of Belo Horizonte, MG, were used as negative controls.

#### 2.2.2. Spiked Blood Samples

Aliquots of peripheral blood samples obtained from 11 healthy individuals were added to the reference sample* L*.* (L*.*) infantum* (MHOM/BR/2002/LPC-RPV) to obtain a concentration of 10,000 parasites/mL of blood.

#### 2.2.3. Evaluation of* kDNA* PCR-ELISA Performance in the Diagnosis of VL, VL-HIV Coinfected, Asymptomatic, and Noninfected Endemic Controls

A minimum sample size of 38 VL cases was calculated with a mean sensitivity of 97.5% to assess the accuracy of the assay, which is consistent with previously published molecular tests [15, 16]. The specificity in these studies was 100%, and a minimum sample size of 38 noninfected controls was estimated with a level of confidence of 95%:

(1) $$\begin{array}{c} n=z^{2}\cdot p\cdot \frac{\left(1-p\right)}{x^{2}}, \end{array}$$

where *p* is the sensitivity or specificity and *x*^2^ is the confidence interval (95%) [17].

The following groups were included.

*Children VL Group*. This group was composed of 81 children admitted to Hospital João Paulo II-FHEMIG (Fundação Hospitalar de Minas Gerais), Belo Horizonte, Minas Gerais, who presented with VL symptoms and parasitological confirmation (direct exam and/or culture) and/or serological diagnosis for VL (indirect immunofluorescence antibody test-IFAT and/or immunochromatographic rK39 antigen-test) associated with clinical improvement after treatment. Patients in this group were aged 3 months to 10 years (mean 2.5 years).

*Adult VL Group*. This group was constituted by 24 adult patients aged 18 to 68 years (mean of 36 years) who were admitted to Hospital Eduardo de Menezes-FHEMIG and presented VL symptoms with parasitological confirmation (direct exam and or culture) and/or serological diagnosis for VL (indirect immunofluorescence antibody test-IFAT and/or immunochromatographic rK39 antigen-test) associated with clinical improvement after treatment.

*VL/HIV Group*. This group was composed of 25 HIV-infected patients who were admitted to Hospital Eduardo de Menezes-FHEMIG, aged 21 to 61 years (mean of 41 years), presenting VL symptoms and parasitological confirmation (direct exam and/or culture) and/or serological diagnosis for VL (indirect immunofluorescence antibody test-IFAT and/or immunochromatographic rK39 antigen-test) associated with clinical cure after treatment. HIV infection was diagnosed by a positive ELISA or chemiluminescence followed by a confirmatory Western blot performed in two different samples.

*Asymptomatic Group*. This group was composed of 33 asymptomatic children, aged 3 months to 6.8 years (mean 2.8 years), living in an endemic area of Belo Horizonte and presenting positive serology (ELISA using recombinant antigen K39 and/or DAT) or molecular (SSU-rRNA qPCR) tests for* Leishmania infection*.

*Noninfected Group*. This group consisted of 40 children, aged 1.1 to 6.7 years (mean 3.5 years), living in an endemic area of Belo Horizonte and presenting negative serological (ELISA using recombinant antigen K39 and/or DAT) and molecular (*SSU-rRNA* qPCR) tests for* Leishmania *spp. infection.

### 2.3. DNA Extraction

DNA from parasites and total DNA from peripheral blood samples and spiked blood samples were extracted using the “QIAamp DNA mini” (QIAGEN GmbH, Hilden, Germany) kit according to the manufacturer's specifications. Negative DNA extraction controls were performed for each experiment by the addition of all reagents except the sample. The yield was determined by absorbance at 260 nm in a spectrophotometer (NanoDrop ND-1000, Thermo Fisher Scientific, Wilmington, DE, USA). The A260/A280 absorbance ratio was analysed to verify the purity of the DNA obtained.

### 2.4. kDNA PCR-ELISA

#### 2.4.1. kDNA PCR Amplification

The reaction was made for a final volume of 25 *μ*L containing 2 *μ*L of DNA sample, 2 units of Platinum Taq DNA Polymerase (Invitrogen, Carlsbad, CA, USA), 1x PCR buffer (200 mM Tris-HCl, pH 8.4, and 500 mM KCl), sense primer 150 (5′-GGGG/TAGGGGCGTTCTC/GCGAA-3′) labelled with biotin at the 5′ end and antisense primer 152 (3′-C/GC/GC/GA/TCTATA/TTTACACCAACCCC-5′) [18] at 0.6 *μ*M each, 2.0 mM MgCl_2_, and 0.2 mM dNTPs (Promega, Madison, WI, USA). The cycling protocol consisted of one step at 95°C for 5 min followed by 34 cycles at 95°C for 30 seconds, 60°C for 30 seconds, and 72°C for 30 seconds. A final step of 72°C for 5 minutes was performed. Each PCR assay included negative (PCR mix without DNA, control for the process of DNA extraction and DNA extracted from samples of healthy volunteers) and positive (genomic DNA extracted from the reference strain of* L. (L*.*) infantum*, MHOM/BR/2002/LPC-RPV) controls.

#### 2.4.2. ELISA Colorimetric Assay

For the detection of the amplified products, the 5′-GATTTCTGCACCCATTTTTC-3′ probe labelled with fluorescein at the 5′ end, designed using the Primer3 program, was used [19]. A better site for annealing of the probe was verified to be among 76 to 91 bp of 120 bp* Leishmania kDNA* fragment amplified using the PCR assay. The search of the lowest homology with genomic sequences from* Leishmania braziliensis*,* Leishmania amazonensis*,* Leishmania guyanensis*,* Trypanosoma cruzi*,* Plasmodium vivax*, and* Mycobacterium tuberculosis* for selection and design of the probe was performed in the BLAST program (National Center for Biotechnology Information website), using the database nucleotides and MegaBlast option.

ELISA followed the parameters described previously [20, 21]. Briefly, wells of MaxiSorp® polystyrene microplates (Nunc Thermo Scientific, Vernon Hills, IL, USA) were coated with 100 *μ*L of streptavidin (5 *μ*g/mL) (streptavidin from* Streptomyces avidinii*, Sigma-Aldrich, St. Louis, MO, USA) in PBS via incubation at 37°C for 1 hour plus overnight at 2–8°C. Coated plates were washed 4 times with PBS-Tween (PBS-T) 0.05%, followed by incubation at 37°C for 2 hours with 200 *μ*L 3% BSA in PBS-T. The plates were washed 4 times with PBS-T and stored at −20°C until use.

PCR at a volume of 100 *μ*L diluted 1 : 25 in PBS were performed in duplicate in microplates at 37°C for 30 minutes. The microplates were washed 3 times with 300 *μ*L of PBS-T and incubated for 10 minutes at room temperature in 0.1 M NaOH 100 *μ*L/well. The microplates were washed once with 0.1 M NaOH (300 *μ*L/well) and 3 times with 300 *μ*L of 0.1 M Tris-HCl. Next, 100 *µ*L/well of a fluorescein-labelled probe at the 5′ end diluted to 0.2 pM in hybridisation solution (70% SSPE5x, 0.75 M sodium chloride, 0.05 M sodium phosphate, 0.005 M EDTA, 30% formamide, and 0.1% SDS) was added and the microplate incubated for one hour at 37°C. The microplates were washed 3 times with 6x SSC solution (0.9 M NaCl and 0.09 M sodium citrate) and twice with a 3x SSC solution (0.45 M NaCl and 0.045 M sodium citrate) containing 0.1% SDS. PBS (150 *μ*L) containing 1% BSA solution (PBS-BSA) was added to each well, and microplates were incubated for 30 minutes at 37°C. An antifluorescein conjugate (antifluorescein/Oregon Green®-peroxidase, Invitrogen-Thermo Scientific, Wilmington, DE, USA) (150 *μ*L) was diluted 1 : 9,000 in PBS-BSA solution and added to each well. The microplates were incubated for one hour at 37°C. The microplates were washed 4 times with PBS-T (300 *μ*L/well) and 100 *μ*L/well of a chromogenic mixture (3,3′,5,5′-tetramethylbenzidine + hydrogen peroxide, Sigma-Aldrich) was added. Plates were incubated for 10 minutes at room temperature in the dark, and the reaction was stopped by the addition of 100 *μ*L of 3N sulfuric acid. Absorbance readings were performed in a microplate reader at 450 nm (Model 550, Bio-Rad, Hercules, CA, USA).

Each ELISA was performed with the positive and negative controls described above for the PCR, and all experiments were performed in duplicate. Data represent the mean values.

#### 2.4.3. *ACTB* PCR-ELISA 

As a control for the DNA extraction and amplification procedure, all samples were amplified for the human beta actin* (ACTB)* gene using an Aco1 (5′-ACCTCATGAAGATCCTCACC-3′) primer labelled with biotin at the 5′ end and an Aco2 primer (5′-CCATCTCTTGCTCGAAGTCC-3′), which generated a product of 120-base pairs [22]. The PCR was performed in a final volume of 20 *μ*L containing 2 *μ*L of DNA sample, 2 units of Platinum* Taq* DNA Polymerase (Invitrogen), 1x PCR buffer (200 mM Tris-HCl, pH 8.4, and 500 mM KCl), 0.5 *μ*M of each primer, 2.0 mM MgCl_2_, and 0.2 mM dNTP (Promega). The cycling protocol consisted of an initial step of 95°C for 5 min and 35 cycles at 95°C for 20 seconds, 60°C for 30 seconds, and 72°C for 1 minute. A final step at 72°C for 6 minutes was performed. Each PCR assay included negative controls (PCR mix without DNA and control for the process of DNA extraction).

The 5′-TCTCCTTAATGCACGCACG-3′ probe labelled with fluorescein at the 5′ end was used to detect the amplified products using the ELISA colorimetric assay [21]. The assay followed the same conditions described above, except that 100 *μ*L of the PCR diluted 1 : 10 in PBS was used, and the antifluorescein conjugate (antifluorescein/Oregon Green-peroxidase, Invitrogen) was diluted 1 : 1,000 in PBS-BSA.

#### 2.4.4. Analytical Precision (Analytical Sensitivity and Specificity)

The detection limit was measured in 6% gel polyacrylamide and kDNA PCR-ELISA for the detection of PCR products from DNA* L. infantum* in the following concentrations: 700 pg, 70 pg, 7 pg, 700 fg, 70 fg, 7 fg, 0.7 fg, 0.07 fg, and 0.007 fg/*μ*L, which were obtained by successive 1 : 10 dilution in RNase-free water. The detection limit was measured via the detection of PCR products from DNA extracted from spiked blood samples with 1, 10, 100, 1,000, and 10,000 of* L. infantum* parasites/mL, obtained by successive 1 : 10 dilutions. The analytical specificity was determined by the analysis of PCR products from genomic DNA extracted of* L. infantum, L. donovani, L*.* braziliensis*,* L*.* guyanensis,* and* L*.* amazonensis* in 6% gel polyacrylamide and kDNA PCR-ELISA.

#### 2.4.5. Diagnostic Precision (Repeatability and Reproducibility)

The intra-assay (repeatability) was performed in four replicates in a single run to detect PCR products amplified from the DNA of the reference* L*.* infantum* (MHOM/BR/2002/LPC-RPV) at 700 fg, 70 fg, 0.7 fg, and 0.07 fg/*μ*L. Four DNA samples from the peripheral blood of patients with VL and four DNA samples from the peripheral blood of noninfected individuals were assayed. Interassay precision (reproducibility) was assessed using PCR products from genomic DNA extracted from* L. infantum*, VL patients, and noninfected individuals, performed in duplicate on consecutive days.

### 2.5. Data Analysis

In the* kDNA* PCR-ELISA development phase, the cut-off was determined using Receiver Operating Characteristic (ROC) curve analysis in GraphPad Prism 5.0 software (San Diego, CA, USA). The detection limit and analytical specificity of the* kDNA* PCR-ELISA were also assessed. The intra-assay (repeatability) and interassay (reproducibility) precision levels were measured using the variation coefficient (VC) of the results of the retested samples using the equation VC = Standard Deviation/Mean × 100.

The sensitivity and specificity were calculated by evaluating the absorbance readings of the* kDNA* PCR-ELISA in 130 DNA samples from VL patients (Children VL Group and Adult VL Group and VL/HIV Group) and 40 samples from the Noninfected Group using ROC curve analyses.

Absorbance readings were individually assessed using the Shapiro-Wilk normality test (*W* test) and compared using the Kruskal-Wallis test. The groups that presented normal (Adult VL and VL/HIV Groups) or nonnormal distribution (Children VL and Asymptomatic VL Groups) were compared using the unpaired *t*-test and Mann–Whitney *U* test, respectively. All tests were performed in the software GraphPad Prism 6.0, considering a level of significance of *p* < 0.05.

The cut-off value for the* ACTB* PCR-ELISA was calculated as the mean of the absorbance readings of the negative controls plus three standard deviations and was determined in the development phase of the assay.

### 2.6. Ethical Considerations

Informed consent was obtained from patients, individuals, or guardians at the time of blood collection and all involved centers evaluated and approved the protocol studies (Protocol Number 14/2011).

## 3. Results

### 3.1. Analytical Sensitivity, Specificity, and Precision of the* kDNA* PCR-ELISA

ROC curve analysis using the absorbance readings of the control samples (14 peripheral blood samples collected from patients with VL and peripheral blood samples obtained from healthy individuals of an endemic area) defined a cut-off of 0.096, which showed better sensitivity without compromising specificity, and an area under the curve of 1.0 (95% CI: 0.86 to 1.0).

**Figure 1:**
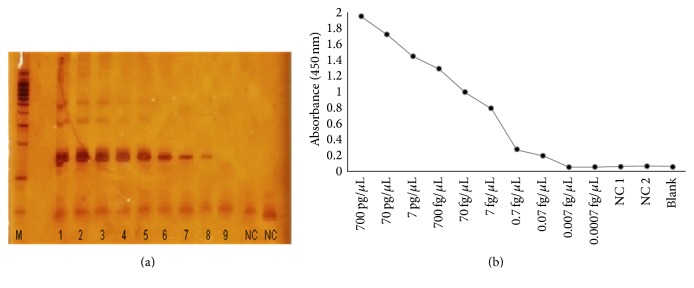
Analytical sensitivity of the conventional PCR and kDNA PCR-ELISA in* L. infantum* gDNA. (a) Analytical sensitivity of 6% polyacrylamide gel and (b)* kDNA* PCR-ELISA for the detection of PCR products from DNA extracted of* L*.* infantum* at concentrations of 1: 700 pg, 2: 70 pg, 3: 7 pg, 4: 700 fg, 5: 70 fg, 6: 7 fg, 7: 0.7 fg, 8: 0.07 fg, and 9: 0.007 fg/*μ*L; NC: negative controls and M: 100 pb DNA Ladder (Promega).

**Figure 2:**
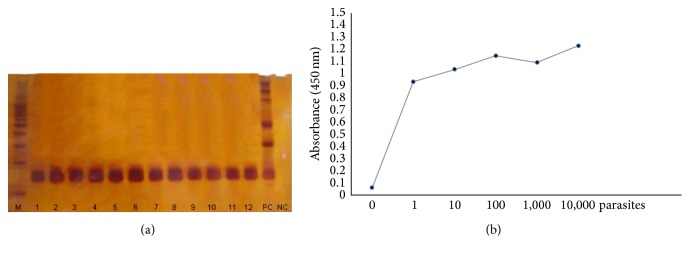
Analytical sensitivity of conventional PCR and kDNA PCR-ELISA in spiked blood samples. (a) Analytical sensitivity of 6% polyacrylamide gels and (b)* kDNA* PCR-ELISA for the detection of PCR products from DNA extracted from spiked blood samples with the addition of 1 parasite (lines 1 and 2) and 10 (lines 3 and 4), 100 (lines 5 and 6), 1,000 (lines 7, 8, and 9), and 10,000 parasites (lines 10, 11, and 12); PC: positive control, NC: negative control, and M: 100 pb DNA Ladder (Promega). The absorbance readings correspond to the mean of the duplicate for each parasite concentration.

This* kDNA* PCR-ELISA presented a limit of detection of 0.07 fg, which was defined using a serial dilution of the reference* L*.* (L*.*) infantum* (MHOM/BR/2002/LPC-RPV) DNA in water. The detection limit was evaluated using DNA extracted from successive dilutions of blood samples (11 samples evaluated) spiked with an initial concentration of 10,000 parasites. The* kDNA* PCR-ELISA consistently detected the lowest concentration: 1 parasite/mL of blood in all samples evaluated. Similar results were observed for electrophoresis analyses on 6% polyacrylamide gels with products from the same PCR assay (Figures 1 and 2).

In the evaluation of analytical specificity, the* kDNA* PCR-ELISA showed positive results for species from the* L. donovani* complex (*L. (L*.*) donovani* and* L*.* (L*.*) infantum*) and negative results for other species (*L. (V*.*) braziliensis, L. (V*.*) guyanensis, *and* L. (L*.*) amazonensis*). Positive results for all species of* Leishmania *were observed when the same products of the PCR assay were analysed in 6% polyacrylamide gel electrophoresis stained with silver nitrate (Table 1).

The obtained variation coefficients (VCs) in the intra-assay tests were 6.81%, 7.78%, 3.44%, and 11.22% for DNA from the culture samples at concentrations of 700 fg, 70 fg, 0.7 fg, and 0.07 fg, respectively, and ranged from 1.36% to 17.55% in the positive blood samples and 2.78% to 8.81% in negative blood samples. The VC values in the reproducibility analyses (interassay precision) were calculated from the results of four different assays performed on consecutive days with the samples evaluated in duplicate. The VCs were 5.36%, 6.03%, 9.44%, and 23.13% for DNA from the culture samples at concentrations of 700 fg to 0.07 fg, respectively, and ranged from 5.44% to 12.8% for positive blood samples and 8.24% to 21.45% for negative blood samples.

### 3.2. Diagnostic Performance of the* kDNA* PCR-ELISA in Different Groups of Patients with VL, VL-HIV Coinfected, Asymptomatic, and Noninfected Endemic Controls

**Figure 3:**
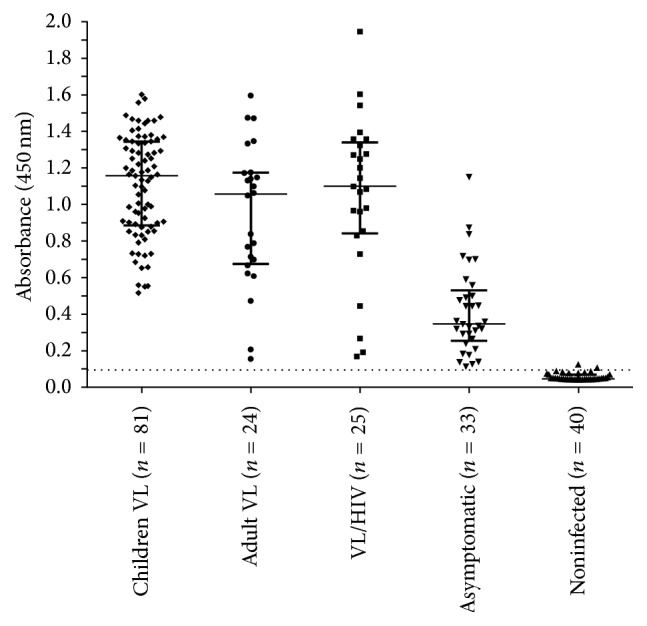
Circulating* L. infantum* DNA levels measured using the* kDNA *PCR-ELISA in different groups.* kDNA *PCR-ELISA absorbance results for the detection of PCR products from DNA extracted from peripheral blood samples in different groups of patients with VL, VL/HIV, asymptomatic, and noninfected endemic controls. There was no statistically difference between Adult VL and VL/HIV Groups (*p* = 0.38). In contrast, the median (0,347) in the Asymptomatic Group was lower than the Children VL Group (*p* < 0.0001). The dashed line represents the cut-off (0.096). Children VL Group: Median = 1.157, 25% Percentile = 0.886 and 75% Percentile = 1.345. Adult VL Group: Median = 1.057, 25% Percentile = 0.675 and 75% Percentile = 1.175. VL/HIV Group: Median = 1.099, 25% Percentile = 0.842 and 75% Percentile = 1.340. Asymptomatic Group: Median = 0.347, 25% Percentile = 0.255 and 75% Percentile = 0.531.

The* kDNA *PCR-ELISA detected infection by* L. (L*.*) infantum* in 100% of the VL Group (children and adult patients) and 100% of the VL/HIV group. The assay detected infection in 100% of asymptomatic individuals living in endemic areas, and only 5% of noninfected individuals (Noninfected Group) had positive results (Figure 3). Therefore, the* kDNA *PCR-ELISA produced an overall sensitivity of 100% (95% CI: 97.1 to 100%) and specificity of 95% (95% CI: 83.5 to 98.6%).

The levels of* Leishmania* DNA measured by the absorbance readings of the PCR-ELISAs were very similar in the Children VL and Adult VL and VL/HIV groups (*p* = 0.18). In contrast, the absorbance readings in the Asymptomatic Group varied from 0.115 to 1.151 (median = 0.347), which indicates lower levels of circulating* Leishmania* DNA in the children of this group (Figure 3).

All samples of the VL, VL/HIV, asymptomatic, and negative groups were assessed using the PCR-ELISA to detect the human* ACTB* gene and produced absorbance readings above the previously determined cut-off (0.099).

## 4. Discussion

Despite the technological developments of the twentieth century, VL diagnosis made little progress in recent decades, with few new or alternative tests. There are two main reasons for this lack of progress: VL is a neglected disease that provides businesses small returns on investment in research and development, and it is an infection with high biological complexity [23].

The present study improves the diagnosis of* L. infantum* in humans by presenting the development and proof-of-concept analysis and phase II evaluation of a* kDNA* PCR-ELISA. This method combines PCR and ELISA into a single analytical technique and provides an objective interpretation of the results compared to conventional PCR followed by electrophoresis because it uses a gene-specific probe to detect the amplified product. The PCR-ELISA also allows multiple sample testing and quantitative and qualitative analyses, such as quantitative real-time PCR (qRT-PCR), but using basic laboratory equipment for the processing ELISA tests that is present in most clinical laboratories [20, 21, 24]. PCR-ELISA has the additional advantage of using a whole blood sample that is obtained in a minimally invasive manner. Recently, Ruiter and colleagues [25] demonstrated that there was no statistically significant difference between the accuracy of molecular methods using whole blood samples in VL diagnosis compared with the use of more invasive bone marrow clinical samples.

The* kDNA* PCR-ELISA is based on the capture of an amplified product with a sense primer labelled with biotin at the 5′end in the polystyrene plate coated with streptavidin, followed by hybridisation with a fluorescein-labelled probe at the 5′ end and an antifluorescein-peroxidase conjugate for detection [21]. Kinetoplast DNA or* kDNA* was chosen as the DNA target for the PCR-ELISA because it is present in a large copy number in the* Leishmania* cell [26, 27]. However, it does not permit precise quantification of the DNA of* Leishmania* spp. because of the large variability in copy number of its minicircle forms (the specific* kDNA* target) in different strains, which led us to choose a qualitative type of a PCR-ELISA.

Different PCR-ELISA were developed for the diagnosis of VL, but most of these assays were evaluated in only one group of patients [28–30], which did not allow broad validation for clinical diagnosis. Other tests were not designed to specifically detect causative agents of VL [28, 29, 31], which discourages its use in surveillance and disease control programmes. Two tests were described only at the development phase [31, 32], and many others used commercial kits for the detection of PCR-amplified products that may suffer from discontinuity of production [28, 30, 33, 34].

The analytical specificity of the* kDNA* PCR-ELISA for causative VL agents was only possible because of the use of a probe designed to complement a specific nucleotide sequence for the* L. donovani* complex. Only the* L. (L.) infantum* and* L. (L.) donovani* species were detected using the assay in the proposed test (Table 1). The same result was obtained using only one platform and a probe specific to* L. (L.) infantum* compared with VL PCR-ELISA previously developed [33].

The lower detection limits of the assay (0.07 fg of purified* L*.* (L*.*) infantum* DNA and 1 parasite/mL of human blood) (Figures 1 and 2) are lower or similar to the detection of other PCR-ELISA [29, 31, 33, 35].

The* kDNA* PCR-ELISA exhibited diagnostic precision because it presented ratios of repeatability and reproducibility from 1.36 to 17.55% and 5.36 to 23.13%, respectively. The upper limit for the coefficient of variation in molecular tests is not established, but our results are similar to other reported tests based on the PCR-ELISA, which revealed intra-assay coefficients of variation (repeatability) from 1.9 to 12.0 and interassay coefficient of variation (repeatability) from 3.0 to 27.0% [21, 36–38]. The same protocol was used to develop the human beta actin* (ACTB) *PCR-ELISA for evaluations of all samples analysed. This test was developed as an internal control of DNA integrity and a control for variation in the efficiency of DNA extraction and PCR amplification primarily in peripheral blood samples that were negative in the* kDNA* PCR-ELISA test. To our knowledge, no other PCR-ELISA that was developed for the diagnosis of VL demonstrated the same type of control.

Phase II evaluation of a* kDNA* PCR-ELISA used 130 DNA samples from the peripheral blood of patients (Children VL Group and Adult VL Group and VL/HIV Group) and 40 samples from a Noninfected Group. The assay exhibited a good performance, with a sensitivity of 100% and specificity of 95%.

The equivalence of* kDNA* PCR-ELISA performance with values demonstrated by other molecular methods for the diagnosis of VL must be highlighted, including PCR assay followed by electrophoresis (sensitivity of 91 to 100% and specificity of 87 to 100%) [15, 16, 39]. The sensitivity of the* kDNA* PCR-ELISA was similar to the value of 98% for the assay developed by Costa et al. and compared with other assays that used the same methodology to diagnose VL and evaluated for test performance [28]. This study used peripheral blood and bone marrow aspirate samples for evaluations, and the DNA target was the* SSU-rRNA* coding region of* L*.* (L*.*) donovani*. The* kDNA* PCR-ELISA demonstrated superior performance compared to the assay developed by De Doncker et al. [29], which used the target* SSU-rRNA* that is conserved in* Leishmania* genus and presented a sensitivity of 83.9% (95% CI: 71.7–92.4) and specificity of 87.2% (95% CI: 72.6–95.7).

The* kDNA *PCR-ELISA was validated in this study in clinical samples from varied groups of VL patients at different ages and asymptomatic and noninfected endemic controls to ensure its widespread use as a method for the diagnosis of the disease and asymptomatic infection. Molecular tests are especially useful for VL diagnosis in immunosuppressed patients, in whom serological tests tend to be less sensitive because of impaired humoral immune response. The sensitivity of* kDNA* PCR-ELISA in VL/HIV coinfected patients was 100%. This result is superior to Piarroux et al. [40] (82%) and similar to Lachaud et al. [41] (100%) (both of these studies used bone marrow samples) and Fissore et al. [42] with serum samples (sensitivity of 97%) and PCR electrophoresis analysis on 2% agarose gel.

Two samples of noninfected individuals (negative group) that previously presented negative results in qPCR were positive in the* kDNA* PCR-ELISA, but with low absorbance readings (Figure 3). This fact may be explained by the molecular target used in the qPCR assay, the* SSU-rRNA*, and these two positive samples may be asymptomatic individuals who were not identified using the reference antibody-based methods. Lachaud et al. [14] compared various molecular targets for the detection of* Leishmania* DNA in peripheral blood samples, the ribosomal DNA target, two nuclear targets, and three kinetoplast (minicircle) targets, and the kinetoplast was generally the most sensitive. Unfortunately, the high number of kDNA copies increases the risk of carryover contamination in the handling and processing steps, mainly if not handled by trained professionals. In the development of kDNA PCR-ELISA all procedures were done by a trained researcher and physical barriers (e.g., separation of rooms and materials and the use of laminar flow hood with ultraviolet light) were utilized in the sample handling steps to minimize the risk of contamination.

Currently, one of the most important challenges in assessing* L*.* infantum* transmission in endemic areas is the identification of asymptomatic infection. Serological tests are the methods of choice for population studies, but our results suggest that these methods are neither adequate nor accurate for the diagnosis of the asymptomatic form. The performance of serological tests to detect* L. infantum* antibodies was evaluated primarily in patients presenting VL symptoms [43–47]. Some studies demonstrated that ELISA methods cannot be reliably used as a tool to identify asymptomatic individuals, and it may underestimate the positive rates, especially when only one antigen is used [48, 49].

The* kDNA* PCR-ELISA in this study is a potential tool to identify and follow up asymptomatic individuals, especially in population-based studies in which there are a larger number of individuals to be evaluated. The assay detected all children from the Asymptomatic VL Group as positive, with absorbance readings from 0.115 to 1.151 and a median (0,347) that was lower than the Children VL Group (Median 1.157; *p* < 0.0001), which suggests the presence of circulating DNA* L. Infantum.* These findings corroborate data published by dos Santos Marques et al. [50], who found lower circulating DNA levels in asymptomatic compared to VL children tested with qPCR targeting SSU-rRNA from* Leishmania* spp. Molecular diagnosis of asymptomatic individuals has great importance because it provides monitoring of individuals who are in contact but have not developed the disease and allows the monitoring of transmission areas to develop more effective strategies for VL control programmes.

## 5. Conclusions

A PCR-ELISA targeting* kDNA* from the* Leishmania donovani* complex was developed for human VL diagnosis in peripheral blood samples. The assay exhibited good performance in proof-of-concept analyses and phase II evaluation in age-variable groups of* L. infantum*-infected patients, asymptomatic individuals, and healthy endemic controls. A human beta actin* (ACTB) *PCR-ELISA was developed as an internal control for DNA extraction and PCR amplification. Therefore, the* kDNA* PCR-ELISA is safe and suitable for the diagnosis of human* L. infantum* infection in reference laboratories in endemic countries and allows the simultaneous testing of a large number of samples with broad application for disease control programmes. It is important to note that the present study is a case control evaluation which may justify the existence of bias and an exaggerated estimate of sensitivity and specificity. So, a large field trial assessing the usefulness of the PCR-ELISA as a confirmatory VL test is still necessary before the PCR-ELISA implementation, both for the evaluation of asymptomatic individuals with positive or negative serology and for the diagnosis of true VL cases among symptomatic suspected patients in an endemic region.

## Figures and Tables

**Table 1:** Analytical specificity of the PCR detected in 6% polyacrylamide gels and the *kDNA* PCR-ELISA using DNA extracted from different *Leishmania* species.

DNA samples	Gel electrophoresis (*kDNA*)	*kDNA *ELISA	
		Absorbance readings (450 nm)	Status
*L. (L.) infantum*	Positive	2,237	Positive
*L. (L.) donovani*	Positive	1,397	Positive
*L. (V.) braziliensis*	Positive	0,056	Negative
*L. (V.) guyanensis*	Positive	0,063	Negative
*L. (L.) amazonensis*	Positive	0,095	Negative
